# Is the online ‘creating healthy eating and active environments survey' (CHEERS) tool reliable for early childhood educators in Alberta, Canada: a randomized crossover trial

**DOI:** 10.1186/s13690-023-01036-z

**Published:** 2023-02-13

**Authors:** Lynne M. Z. Lafave

**Affiliations:** Department of Health and Physical Education, Mount Royal University, Calgary, AB T3E 6K6 Canada

**Keywords:** Early childhood education, ECEC, eHealth, Online, Nutrition, Healthy eating, Physical activity, CHEERS, Audit tool, Reliability

## Abstract

**Background:**

The creating healthy eating and active environments survey (CHEERS) is an audit tool used to assess the nutrition and physical activity environment in early childhood education and care (ECEC) centres. Availability of the tool has been limited to paper-based versions. Digital health initiatives offer improved reach and immediacy of support for community-based clients through novel technology products. In order to provide increased access to the CHEERS tool, an online version was developed. The objective of this study was to assess the reliability of an online version of CHEERS.

**Methods:**

Utilizing a randomized crossover design, ECEC educators completed either a paper-based or online-based survey and then the opposite mode with a two to three-week interval. The intraclass correlation coefficient (ICC, with 95% confidence interval) was used to determine the reliability between test and retest. Absolute index of reliability in the original measurement was assessed through the standard error of measurement (SEM = SD × √1-ICC). The smallest amount of change not due to inherent variation was assessed by determining minimal detectable change at the 95% confidence level (MDC_95_ = SEM × 1.96 ×√2; MDC_95_% = MDC_95_/mean ×100).

**Results:**

Test–retest reliability was good to excellent for the online-based CHEERS total score (ICC = 0.86) and for each of the four subscales: food served (ICC = 0.82), healthy eating environment (ICC = 0.76), program planning (ICC = 0.76), and physical activity environment (ICC = 0.79). The SEM, MDC_95,_ and MDC_95_% for the CHEERS overall score were 0.79, 2.19, and 9.6%, respectively.

**Conclusions:**

The results of this study demonstrate that the online-based and paper-based versions of the CHEERS audit tool share comparable accuracy. The CHEERS tool can be reliably implemented in an online environment and this provides users an alternative means to complete the centre-based health assessment. The advantage of the online-based version includes user accessibility and the potential to develop a feedback response for participants using digitally collected data.

**Supplementary Information:**

The online version contains supplementary material available at 10.1186/s13690-023-01036-z.

## Background

The early years are a critical period in child development where the origins of lifestyle health behaviours can be identified. Patterns of eating and activity, as early as one to two years of age, have been demonstrated to track through childhood and into adulthood [1–6]. Research suggests that Canadian preschool-aged children consume less than one-third of the vegetable and fruit intake recommended combined with low levels of physical activity [7–10]. It is estimated that close to 60% of Canadian children 0–5 years participate in some type of childcare arrangement with over half of these attending centre-based care [11]. This demonstrates that early childhood education and care (ECEC) environments represent rich opportunities for interventions that promote healthy eating and reduced sedentary behaviours aimed at preventing childhood obesity and the reduction of the long-term non-communicable chronic disease [12–14].

Supporting child health and well-being is a public health priority and is an essential global health initiative. The nurturing care framework is an organizing principal that structures key components of high quality care in ECEC environments that is composed of five elements: health, nutrition, security and safety, responsive caregiving, and early learning opportunities [15]. In order to provide this high quality care, educators and public health professionals require access to valid and reliable tools. The creating healthy eating and active environments survey (CHEERS) tool is a community-based, educator-administered audit tool designed to offer ECEC centres an evaluative measure to assess their centres healthy eating and physical activity environment. The constructs measured include the quality of food served, the environment where children are eating, the curriculum connections for nutrition education, and the physical activity opportunities provided. This will also be a valuable tool for public health providers and researchers that can be used to measure the environmental context in a child care setting. The CHEERS tool can facilitate and empower ECEC centres to enhance nutrition and activity environments that support child health. While currently aligned with Canadian best practices, the tool would be adaptable to many international childcare contexts.

The tool has been assessed for reliability and validity with early childhood experts and educators. The CHEERS tool was content validated following a structured process involving a panel of ten nutrition, physical activity, early childhood, and public health professionals with a readability of grade 8.1 using Flesch–Kincaid [16] with evidence of good inter-rater reliability, intra-rater reliability, internal consistency, and concurrent validity [17].

The CHEERS tool was developed as a paper instrument that could be completed by an educator and returned by ground mail for evaluation. Paper-based questionnaires are human resource intensive and introduce the possibility of data omission and errors in data transcription to digital format. On the other hand, there are disadvantages of online-based questionnaires such as inaccurate responses due to misinterpretation of questions and low response rates. The internet and its applications are a continuous area of innovation and an exponentially increasing component of every day life. Electronic data collection is becoming more extensively used in healthcare research that bring with it many advantages such as access and service provision for remote communities, facilitated data entry time, reduced processing cost, and faster survey response return to stakeholder [18, 19]. Moreover, a key message from the early childhood community is the need for reciprocation. While data is collected from the ECEC centre, how can the centre benefit from completing the questionnaire? Educator awareness of policies and practices that support or impair healthy environments for children have been identified as a method of informing or reaffirming health promoting professional practice activities [20].  An online-based survey can increase response time and support just-in-time learning for educators.

An electronic version of the CHEERS tool was developed to align with increasing use of electronic public health outreach and resources provided through website in addition to the desire of educators to access the tool in an online context. While reliability and validity of the CHEERS tool have been demonstrated, it is not known if administration using an online-based interface is consistent with paper and pencil versions. The aim of this study was to assess the reliability and utility of an electronic version of the CHEERS tool.

## Methods

This study was conducted in Alberta, Canada throughout provincial health zones. Ethical approval to conduct the study was obtained from the Mount Royal University Human Research Ethics Board (no. 100016). Participants were fully informed about the purpose and procedures of the study and provided written consent for paper first participation or digital consent for electronic first participation. No minors were involved in this study.

### Participants

The intended users of the CHEERS tool, ECEC educators, were recruited from licensed ECEC facility-based centres that care for infants, toddlers and pre-school-aged children in Alberta, Canada. They typically provide care throughout the day, from the morning to early evening. To be eligible for the study, ECEC educators had to be employed full time in licensed ECEC centres that provided care for a minimum of 15 preschool aged (3 – 5 years) children with the classification of day care program. Inclusion criteria was access to an electronic device (smartphone/tablet/laptop), no previous experience with the CHEERS survey, and fluent in English. Exclusion criteria were exclusive employment in family day home or after school care programs or employment in unlicensed ECEC centres.

### CHEER audit tool

The CHEERS audit tool includes 59 items divided into four subscales: food served (23 items), healthy eating environment (18 items), healthy eating program planning (6 items), and physical activity environment (12 items). Response options for each item range from always to never which are then recorded from 1 to 7. Scores are averaged for each subscale and summed for the CHEERS total score (range 4–28). A copy of the questionnaire is available [21] and included as a supplemental file (see Supplemental file 1).

### Study design

This study is a randomized crossover trial design comparing paper and electronic survey formats. A convenience sample of ECEC educators were recruited from ECEC centres throughout Alberta. Flyer advertisements were distributed provincially through email, inviting ECEC educators to contact the project coordinator through email. A $20 gift card was provided to respondents as an incentive. Interested individuals were contacted via email or telephone and given detailed instructions about the study to ensure sample validity and data integrity [22]. Educators were randomly assigned by random generated number to complete the CHEERS survey either in paper version or electronic survey platform. The two study groups were reversed according to the randomized crossover design and the alternate mode completed within a two to three-week interval. The interval period being sufficiently short to avoid real change and sufficiently long enough to minimize recall with 2 weeks being a common interval in the development of health measurement scales [23].

### Study methods

Participants assigned to paper-first administration received the CHEERS survey in a sealed envelope with instructions on how to complete the survey as well as a prepaid pre-addressed envelope to use to return the paper survey to the research team. An email reminder was sent to the participant ten days after mailing to ensure participants received the survey and to encourage return of the survey. In three cases, the paper surveys had not arrived and an additional package had to be resent. Upon return, surveys were coded and data entered into a Microsoft Excel 2016 spreadsheet. In the case of missing data, the input was left blank. If a page of survey responses were missed the survey was considered spoiled and the participant considered lost to follow up. To implement the washout period in the paper-first group, the participant was sent a personalized email link to the online version of the CHEERS survey one week after receiving the paper survey (three business day delivery within province). A reminder email was sent one week after the first email to those individuals where no recorded survey was evident in the online platform. Participants assigned to the online-first administration received a personalized the link to the online version of CHEERS which contained instructions on how to complete the survey and was followed by a reminder email one week later. To implement the washout period in the online-first group, the paper survey package was mailed to participants one week after the survey was verified as received in the online platform. An email reminder was sent to participants ten days later to check for paper-survey receipt and encourage return. In two cases, paper surveys had to be resent and in three cases participants indicated surveys were mailed back however these were never received by the research team.

### Online interface

**Fig. 1:**
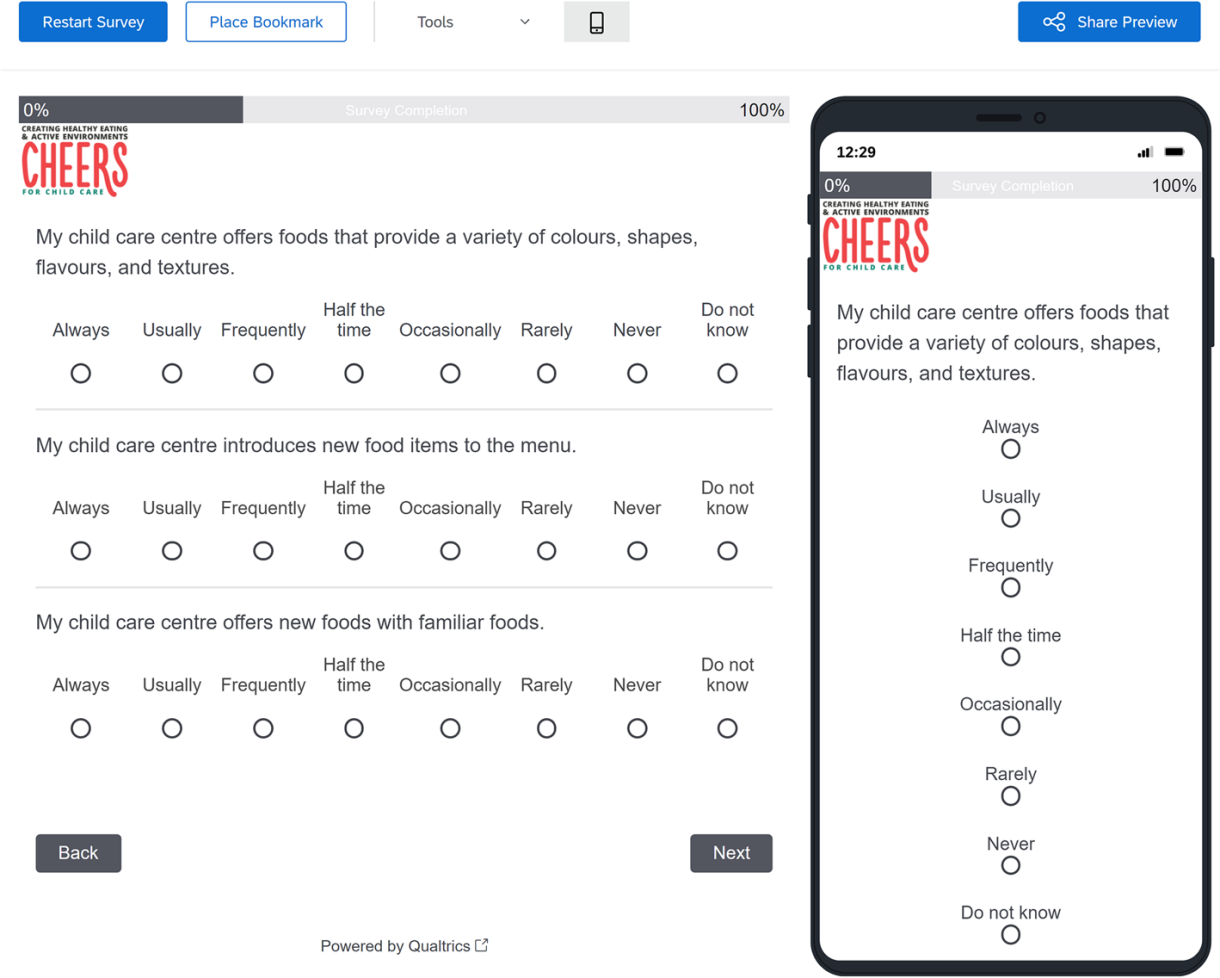
Screenshot of the electronic CHEERS tool as it appears in a computer browser (explorer, left) or a mobile device (right)

The electronic questionnaire system for the CHEERS administration was built in Qualtrics® online-based survey platform. This survey system is compatible across computer and mobile smartphone platforms (e.g. Apple Safari, Google Chrome, Internet Explorer, Mozilla Firefox). The survey is optimized to the mobile experience to adjust for screen size and formatting. The online version of the CHEERS questionnaire was developed to replicate the paper-based questionnaire experience as closely as possible. The online questionnaire presents three questions per page with a progress tracking bar at the top of the survey. The survey platform also records the duration (time to complete the survey). A sample screenshot of the online-based interface for the survey tool is presented in Fig. 1. Participants were provided unique email link once identity and eligibility verified.

### Sample size

Sample size needed for the reliability analysis was calculated using the sample size calculator [24] using the parameters of required number of participants when β = 0.20, α = 0.05, the number of repetitions per participant as two, with a potential dropout rate of 30%. We chose an ICC = 0.70 as minimally acceptable and an ICC = 0.90 as desired based on the recommended minimum reliability of 0.70 when the scale is used in research [23]. This calculation returned a required sample size of 23 participants per group (paper 1^st^ or online 1^st^) or 46 participants estimated to be sufficient for testing reliability [23–25].

### Statistical analysis

The results of the paper questionnaire were manually inputted into Microsoft Excel 2016 by the research assistant and data checking completed by the PI. Online data was captured by the Qualtrics software program and derived directly from the system database. Statistical analysis was performed using SPSS statistical package version 26 (SPSS Inc, Chicago, IL), with alpha level of *p* < 0.05. Descriptive statistics were used to report means and variation between trials for the overall CHEERS score and subscales. Group comparisons were performed using independent t-test and Pearson chi-square tests for numerical and categorical demographic variables, respectively, to assess heterogeneity between ECECs. Interquartile range (IQR) and Mann–Whitney U test was used to assess survey duration (time to complete the survey) in the online-based environment.

To assess the magnitude of the association between electronic and paper data, the intraclass correlation coefficient (ICC) was calculated by SPSS for each administration and administration time points along with their 95% confidence interval. The ICC takes into account the selection of raters as well as the correlation and agreement between raters [26–28]. The intraclass correlation coefficient estimates and their 95% confident intervals for test–retest reliability were based on a single rater/measurement, absolute-agreement, 2-way mixed-effects model [26, 29]. In this study, interpretations of ICC results followed Koo and Li’s categorizations: poor (less than 0.5), moderate (0.5–0.75), good (0.75–0.9), and excellent (greater than 0.90) [21]. To further assess response stability, the standard error of measurement (SEM) was calculated using the following formula: SEM = SD × √(1 – ICC) [30, 31]. The SEM provides an index of the “trial-to-trial noise in the data” which provides an absolute index of reliability in the original measurement unit without the influence of variance among participants [30]. Smaller SEM values, relative to the unit of measure of the score, demonstrate lower response variation from test to retest. The SEM value might be considered an estimation of the expected random variation in scores when no real change has taken place. Absolute reliability provides additional information about a change in score and what change would be required to provide a measure of true change beyond expected measurement error and individual variabilities. This can be evaluated using the minimal detectable change (MDC_95_) which provides a value of reliability in the original units of the measure reflecting the amount of change required to demonstrate a difference not due to chance at the 95% confidence level [32]. MDC_95_ was calculated using the following formula: MDC_95_ = SEM X 1.96 X √2 [33]. This change can be expressed as relative change (MDC95%) and was calculated in this study using the following formula: MDC_95_% = MDC_95_/mean X 100 [33]. An MDC_95_% less than 30 has been interpreted in the literature as acceptable random measurement error and under 10 as excellent [34, 35]. Utility was evaluated by documenting the length of time taken to complete the CHEERS tool as recorded in the survey platform housed in Qualtrics [36].

## Results

### Participants

**Fig. 2:**
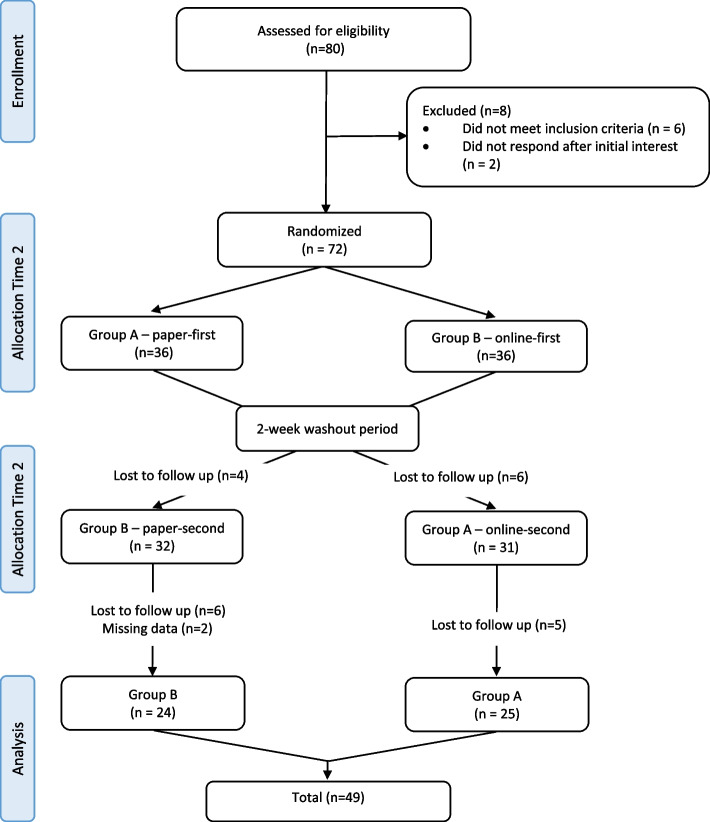
Study design and recruitment

A total of 80 educators aged between 26 and 55 + years volunteered to participate in the study (Fig. 2). Eight educators did not meet inclusion criteria or did not respond after initial interest and were excluded from randomization and the final 72 randomly assigned to a survey platform. Eleven participants did not continue in the paper-first survey (group A) and twelve in the online-first survey (group B). Two surveys in group B were excluded due to missing multiple survey questions on double sided page. Missing cases were excluded when the questionnaire scores were compared.

### Participant demographics

Demographic characteristics of the child care centres are presented in Table 1. ECEC centres were representative of both not-for-profit and profit-based programs. Half of participating ECEC centres were from large urban centres with the remaining a mix of medium and small centres. No statistically significant differences among demographic characteristics of child care type (χ^2 ^[1] = 1.647, *p* = 0.199), city size (χ^2 ^[2] = 3.163, *p* = 0.206), or number of preschoolers enrolled (t [47] = 0.452, *p* = 0.145) between the two groups were noted.

**Table 1:** Licensed early childhood education centre characteristics, Alberta, Canada

	**Group A Paper 1**^**st**^ (*n* = 25)		**Group B Online 1**^**st**^ (*n* = 24)	
	*n*	%	*n*	%
**Childcare type**				
Not-for-profit	15	60	10	40
For-profit	10	40	14	60
**City size**				
Large urban population centre	12	48	10	40
Medium population centre	5	20	10	40
Small population centre	8	32	5	20
**Number of preschoolers enrolled (M, SD)**	29.3	23.5	32.0	17.3

Demographic characteristics of the child care educators are presented in Table 2. All respondents were female with representation throughout educator training levels. Educator demographics were similar between the groups in terms of age (χ^2 ^[4] = 3.667, *p* = 0.453), level of education (χ^2 ^[2] = 0.091, *p* = 0.956), or years of experience (t [47] = 0.278, *p* = 0.951).

**Table 2:** Early childhood educator characteristics, Alberta, Canada

	**Group A Paper 1**^**st**^ (*n* = 25)		**Group B Online 1**^**st**^ (*n* = 24)	
	*n*	%	*n*	%
Gender				
Female	25	100	24	100
Male	-	-	-	-
Educator age				
26–35 yrs	2	8	7	28
36–45 yrs	8	32	7	28
46–55 yrs	8	32	4	16
55 + yrs	4	16	3	12
Prefer not to answer	3	12	4	16
Education background				
Certificate	6	24	7	28
Diploma	14	56	14	56
University degree	6	20	4	16
**Number of years ECEC Experience (M, SD)**	10.4	8.2	9.7	8.2

### Reliability

Overall test–retest reliability of form (paper versus online) and sequence (paper first versus online first) was assessed using the ICC two-way mixed-effects model and absolute-agreement. The ICC values for overall CHEERS score and subscales were good to excellent (0.77—0.92) regardless of form (paper vs online) or administration sequence (Table 3).

**Table 3:** CHEERS tool scores and intraclass correlation coefficient measures of test–retest reliability between form (online vs paper) and sequence (online first or paper first)

**Group A—Paper 1**^**st**^				
	**Paper *n* = 25** **Mean (SD)**	**Online *n* = 25** **Mean (SD)**	**ICC**	**95% CI**
CHEERS overall	22.2 (2.8)	22.9 (3.1)	0.91	0.79–0.96
FS	6.0 (0.5)	6.0 (0.7)	0.91	0.80–0.96
HEE	6.2 (0.6)	6.1 (0.8)	0.81	0.57–0.92
HPP	4.5 (1.1)	5.0 (1.2)	0.77	0.44–0.90
PA	5.5 (0.9)	5.8 (0.9)	0.92	0.74–0.97
**Group B—Online 1**^**st**^				
	**Online *n* = 24** **Mean (SD)**	**Paper *n* = 24** **Mean (SD)**	**ICC**	**95% CI**
CHEERS overall	22.8 (2.7)	23.2 (2.6)	0.93	0.84–0.97
FS	5.9 (0.5)	6.2 (0.5)	0.83	0.25–0.94
HEE	6.2 (0.6)	6.2 (0.5)	0.93	0.83–0.97
HPP	4.9 (1.3)	5.0 (1.3)	0.93	0.84–0.97
PA	5.9 (0.8)	5.8 (0.8)	0.89	0.75–0.95

*FS* Food served, *HEE* Healthy eating environment, *HPP* Healthy program planning, *PA* Physical activity

Evaluation between time 1 and time 2 shows good to excellent reliability (Table 4). The ICC value between administration for the overall CHEERS score was 0.92 (95% CI: 0.84–0.96). ICC values for the subdomains ranged 0.85–0.90.

**Table 4:** CHEERS tool scores and intraclass correlation coefficient measures of test–retest reliability between Allocation Time 1 and Time 2

	**Time 1 *n* = 49 Mean (SD)**	**Time 2 *n* = 49 Mean (SD)**	**ICC**	**95% CI**
CHEERS overall	22.5 (2.7)	23.1 (2.8)	0.92	0.84–0.96
FS	5.9 (0.5)	6.1 (0.6)	0.87	0.72–0.93
HEE	6.2 (0.6)	6.2 (0.7)	0.85	0.74–0.92
HPP	4.7 (1.2)	5.0 (1.2)	0.86	0.72–0.92
PA	5.7 (0.9)	5.8 (0.8)	0.90	0.83–0.95

*FS* Food served, *HEE* Healthy eating environment, *HPP* Healthy program planning, *PA* Physical activity

### Agreement

The SEM, MDC_95_ and MDC_95_% of the CHEERS scores and subdomain tests are shown in Table 5. The SEM between administration of surveys (time 1 versus time 2) were relatively small for the CHEERS overall score (0.79) and subdomains (0.21 – 0.45) demonstrating an acceptable level of agreement between time points.

**Table 5:** Reliability and minimal detectable change values for overall CHEERS tool scores

	***SEM***	***MDC***_***95***_	***MDC***_***95%***_
CHEERS overall	0.79	2.19	9.6
FS	0.21	0.57	9.5
HEE	0.25	0.69	11.1
HPP	0.45	1.26	25.8
PA	0.26	0.72	12.6

*FS* Food served, *HEE* Healthy eating environment, *HPP* Healthy program planning, *PA* Physical activity, *SEM* Standard error of measurement, *MDC*_*95*_ Minimal detectable change at 95% confidence interval, *MDC*_*95*_*%* Percent minimal detectable change at 95% confidence interval

The MDC_95_ of the overall CHEERS score, food served, healthy eating environment, program planning, and physical activity were 2.19, 0.57, 0.69, 1.26, and 0.72, respectively. The MDC_95_% in the overall CHEERS score, food served, healthy eating environment, program planning, and physical activity were 9.6%, 9.5%, 11.1%, 25.8%, and 12.6%, respectively.

### Utility measure

The median time taken to complete the CHEERS tool for all educators, regardless of sequence, in the online environment was 16.6 min (IQR 12.7–29.4). The median time taken to complete the survey in an online-first sequence (Group B) was 12.3 min (IQR 12.3–33.8). The median time taken to complete the survey in an online-second sequence (Group A) was 16.1 min (IQR 12.7–19.6). A Mann–Whitney U test revealed that time taken to complete the study was not significantly different between online-first (Group B, Md = 12.3, *n* = 24) compared to the online-second sequence (Group A, Md = 16.1, *n* = 25), U = 254.5, *p* = 0.363.

## Discussion

The results of this study demonstrate that the online version of the CHEERS tool presents good reliability, agreement, and utility to its paper version. Previous research demonstrates that the paper-based, educator-administered CHEERS survey is a reliable and valid audit tool for evaluating child care centre eating and activity environments [16, 17]. Electronic health (eHealth) and mobile health (mHealth) innovations offer new avenues for health promotion [37]. Online methods of health assessment and interventions have the advantages of convenience, ease of operation, adaptability, and accessibility. However, evidence of online reliability, validity, and effectiveness are an important component of building comprehensive evidence for the eHealth initiative [38].

The test–retest reliability of the online-based CHEERS overall score was strongly aligned with the paper-based administration (ICC = 0.91).

These results align well with previously reported paper-based reliability data for the overall score (ICC = 0.81) [17]. Psychometric properties of “r*eliability and agreement are estimates that vary based on interactions between a tool, its user, and the context of the assessment”* [39]. Considering that the current study was done with a new cohort of early childhood educators who had not previously participated and similar intra-rater results were found contributes to the overall psychometric evidence demonstrating reliability of the CHEERS audit tool.

The outcomes of this study are in accordance with outcomes in similar paper to online validation studies of child related health tools. For example, the NutriSTEP® questionnaire screens nutrition risk in preschoolers through a survey completed by primary caregiver/parents. An ICC of 0.91 for CHEERS overall score is in line with the ICC of 0.91 found in the NutriSTEP® adaptation from paper-based to online-based administration [40]. There was similar alignment of ICC scores for the MiniPAQLQ, a quality of life questionnaire completed by caregiver’s of children with asthma, ICC of 0.89 [41]. This is further supported by the results of a meta-analysis suggesting that computer and paper-based questionnaire administrations result in equivalent scores when comparing electronic and paper-based administrations [42].

The standard error of measurement (SEM) is a measure of stability, reflecting the precision of individual scores expressed in the same units as the original scores [23, 30]. While ICC reflects reliability among people, SEM quantifies precision within individuals and permits the calculation of the minimal detectable difference that can reliably meaningful difference to measure change that can be used in future research to determine if an intervention has a significant impact on creating change. The current study has provided evidence of stability as demonstrated by the SEM, MDC_95_, and MDC_95_% from paper-based to online-based administration.

Tool reliability and validity are important aspects of health measurement scales. However, a measure must also have utility and ease of use for respondents thus an important component of psychometric tool assessment is the usability, functionality, and availability of the tool [43, 44]. The literature clearly indicates a preference for digital over paper questionnaire completion [40, 41, 45]. eHealth technologies can provide improved efficiency and accessibility for health promotion services for stakeholders. This study adds to the reliability and utility of the CHEERS tool through the addition of a user-friendly electronic version.

Potential limitations exist with this research. First, all participants in this study were women which brings into question the representativeness of the study sample. However, the fact that the ECE workforce is comprised primarily of women, 96% of all ECEs in Canada are women [46], this sampling does reflect the population of users intended for this tool. Second, findings may also be limited because this study used a convenience sample. While a random sampling frame would be a better approach, the early childhood educator profession in Canada does not have a central professional community where all members are listed. As a result, there is no mechanism from which to randomly select participants. The provincial government website has a listing of all licensed centres throughout the province by region so emails to centres provide the best option to contact a variety of educators in different cities and towns of various sizes. Thirdly, there is a possibility that participants remembered their responses from the first administration. A recommended interval between administrations appropriate for reliability studies assessing the development of health measurement scales is approximately 2 weeks [23]. Sufficiently short to ensure minimal opportunity for real behaviour change while sufficiently long enough to minimize recall and bias. However, some individuals may have remembered their responses and which may have allowed them to report similar answers in the second administration. Lastly, a limitation is the dropout rate. Thirty-six participants were assigned to arms of the crossover study, however, approximately ten were lost to follow-up resulting in a 27% dropout rate which within the 30% dropout rate [23]. This loss may have been due to characteristics of the survey (time demand) or high job turnover in the early childhood educator workforce [47, 48]. Five paper surveys were lost to lack of postal delivery (lost mail) and two lost due to incomplete data (missed backside of paper-survey). However, the reliability measures of the CHEERS adaptation from paper-based to electronic-based administration were similar the NutriSTEP® and MiniPAQLQ paper-to-electronic adaptations within a similar population and it is reasonable to assume the missing data would not have meaningfully impacted the study outcome.

## Conclusions

This study provides evidence of good test-rest reliability of the online-based CHEERS tool relative to the paper-based version. Health interventions are increasingly being made available via multiple platforms (computer or mobile versions) and demonstrating that these digital options reliable improves the reach and immediacy of support for community-based clients through novel technology products. eHealth administration of the CHEERS tool can be used in public health initiatives within community settings and the outcomes can be interpreted in accordance with findings of the paper version of the tool. Future opportunities would be to investigate immediate feedback of the survey to respondents as well as educational outreach.

## Supplementary Information

**Additional file 1**.

## Data Availability

The raw datasets are not publicly available as permission for sharing this dataset or making it publicly available was not requested as part of the application to the Research Ethics Board and participant consent for public sharing of their data not secured.

## Abbreviations

CHEERS: Creating healthy eating and activity environments survey
CI: Confidence interval
eHealth: Electronic health
ECEC: Early learning and childcare
FS: Food served
HEE: Healthy eating environment
HPP: Healthy eating program planning
ICC: Intraclass correlation coefficient
MDC_95_: Minimal detectable change at 95% confidence interval
MDC_95_%: Percent minimal detectable change at 95% confidence interval
PA: Physical activity environment
SEM: Standard error of measurement
